# Miscellany

**Published:** 1899-06

**Authors:** 


### CHRISTIAN SCIENTISTS IN THE TOILS.

AS THE JOURNAL goes to press there is being held before United States Commissioner Robinson an examination in a Christian science case which bids fair to become celebrated. George H. Kinter and his wife, who are healers in the Christian science church, and James Saunders and his wife have been arrested by the United States authorities on charge of manslaughter.

Mr. and Mrs. Saunders have been living at Fort Porter with Captain and Mrs. Sample, Captain Sample being an officer of the 13th Regiment. The Saunders's 7 year old son, Ralph Lamont Saunders, died the morning of the 22d. At his last breath Dr. Walter D. McCaw, post-surgeon, was called in. He refused to certify to death and reported to the coroner. The result was a post mortem which showed the child to have died of double pneumonia. It was learned that the Kinters had been "treating" the child, and the arrests followed. The evidence so far has shown that although Dr. McCaw, post-surgeon and Dr. Nelson W. Wilson, assistant surgeon, were at Fort Porter all the time, they had not been called.

District-attorney Emory P. Close is conducting the prosecution, and associated with him are his assistant Mr. Brown, and Mr. Tracy C. Becker, representing the Erie County Medical Society. Dr. William H. Heath is associated with the prosecution as medical expert. The entire moral, social and financial force of the Christian scientists has been thrown into the fight, which promises to be a long one.

---

### ADDITIONAL PERSONAL.

APPOINTMENTS to the Sisters of Charity Hospital resident-staff have been announced as follows: Dr. William T. Griffin, senior physician; assistants, Dr. Martin Downey, Dr. Henry L. Siedler, Dr. Bernard F. Dennis, of the University of Buffalo, and Dr. W. C. Gerber, of Denver.

